# Clinical Effective Dynamic Range and the Measurement Floor of SITA-Faster Visual Field Tests

**DOI:** 10.1007/s44402-026-00103-6

**Published:** 2026-05-11

**Authors:** Jack Phu, Henrietta Wang, Michael Kalloniatis

**Affiliations:** 1 School of Optometry and Vision Science, University of New South Wales, Kensington, New South Wales Australia; 2 University of Houston College of Optometry, Houston, Texas USA; 3 Faculty of Medicine and Health, University of New South Wales, Kensington, New South Wales Australia; 4 School of Medicine (Optometry), Deakin University, Waurn Ponds, Victoria Australia

**Keywords:** 24-2, Frontloaded, Perimetry, Standard automated perimetry, Visual fields

## Abstract

**Purpose:**

To describe the effective dynamic range (defined below) using the Swedish Interactive Thresholding Algorithm (SITA)-Faster perimetry and the effects of reliability.

**Methods:**

Nine thousand six hundred and seventy-two test–retest pairs of the 52 test locations of the 24-2 SITA-Faster visual field tests from 1468 eyes of 748 subjects were analysed. The outcome measures were the number of discernible clinical steps, breakpoints and the measurement floor. Effective dynamic range was first assessed using an established approach of smoothed Loess functions (Method 1) applied to test–retest sensitivity pairs. Method 1 was a geometric approach for estimating the outcome measures. Method 2 used the same Loess functions applied to the data in Bland–Altman form. In Method 3, Gaussian smoothing and multisegmental linear regression were applied to the standard deviation of test–retest pairs as a function of mean sensitivity. Analyses were performed on the total cohort, a false positive rate ≤15% subgroup and a false positive rate 0% subgroup.

**Results:**

Method 1 returned four clinical steps with intervals bounded by 37 dB, 27 dB and 20–21 dB (the measurement floor), similar across each reliability condition. Method 2 returned the same number of clinical steps, but with a lower measurement floor (18–19 dB). Method 3 led to one to two additional steps and a floor of 18–21 dB. In general, using a false positive rate of 0% resulted in a relatively lower measurement floor value and more breakpoints in comparison with higher false positive rate tolerances.

**Conclusions:**

Using a conventional reliability criterion of false positive rate ≤15%, SITA-Faster has four to five meaningful clinical steps/intervals and a measurement floor of 18–21 dB, which is slightly higher than estimates from SITA-Standard (likely attributable to the higher sensitivity values and greater variability of SITA-Faster). Below this level, clinicians should consider other perimetric approaches, such as 10-2 and/or testing with a Goldmann size V stimulus.

Key Points

- Swedish Interactive Thresholding Algorithm (SITA)-Faster perimetry has four to five meaningful clinical steps/intervals.
- SITA-Faster perimetry has a measurement floor of 18–21 dB, which is slightly higher than estimates seen in the SITA-Standard methodology.
- The dynamic range of sensitivities may also be slightly lower in SITA-Faster compared with SITA-Standard.

## Introduction

Perimetry is a cornerstone of assessing visual function, especially in the diagnosis and monitoring of diseases of the visual pathways [1]. In current clinical practice, the clinical standard approach for quantifying the visual field is using projection-based systems, including perimeters such as the Humphrey Field Analyzer (Carl Zeiss Meditec, zeiss.com/meditec-ag/en/home.html), Medmont Perimeter (Medmont International Pty Ltd, medmont.com.au/), Octopus Perimeter (Haag-Streit, haag-streit.com/), Henson (Topcon Healthcare, Inc, https://topconhealthcare.com/) and others [2]. Most commonly, perimetry using these systems involves the presentation of an increment white light stimulus from an external light source upon a uniformly illuminated white background [2]. The measure of differential light sensitivity (the contrast between stimulus and its background) is conventionally conveyed using a decibel (dB) scale, representing the level of attenuation of the stimulus at which it is near the threshold of perception [1].

Therefore, perimeters have a physical dynamic range over which contrast sensitivity can be quantified. This is computed using the maximum luminance output (i.e., maximum stimulus intensity) and the background. For example, the Humphrey Field Analyzer, with its maximum luminance output of 3413 cd m^−2^ and background luminance of 10 cd m^−2^ has a physical range of 0–50 dB.

However, the physical dynamic range is different to the clinically meaningful dynamic range for the subject. Test–retest variability inherent to the subject and test means that clinically meaningful differences are different from the physical increments presented by the device [3]. Understanding test–retest variability and dynamic range is important for identifying significant deviations from normality, or intra-patient change in visual function [3].

Previous studies have examined the dynamic range of clinical perimetry using older test algorithms. Wall et al. [4] showed that the clinically meaningful dynamic range of the Humphrey Field Analyzer using a Goldmann size III stimulus and Swedish Interactive Thresholding Algorithm (SITA) Standard testing algorithm comprised four steps with a measurement floor of 15–19 dB. Gardiner et al. [5] found a similar measurement floor when correlating SITA-Standard measurements with Method of Constant Stimuli results.

More recently, SITA-Faster has become increasingly used in clinical practice [6, 7]. Previous studies have shown that SITA-Faster is likely to return more variable results compared with SITA-Standard [4, 8]. Additionally, SITA-Faster results in higher false positive rates compared to SITA-Standard, which may be a correlate to higher sensitivity levels [8]. Accordingly, the algorithmic and measurement differences between SITA-Standard and SITA-Faster may lead to differences in discernible test intervals and measurement floor.

The purpose of the present study was to use intra-visit repeated (‘frontloaded’) [9] SITA-Faster visual field results to examine the effective dynamic range (discernible measurement intervals, their breakpoints and the measurement floor). The effect of different false positive criteria on the dynamic range was also examined.

## Methods

### Ethics Statement

This was a retrospective, cross-sectional study performed in line with the principles of the Declaration of Helsinki. Approval was granted by the Human Research Ethics Committee of the University of New South Wales (HC210563). The study adhered to the tenets of the Declaration of Helsinki. Informed consent was obtained from all individual participants included in the study.

### Visual Field Testing

Data were obtained from patients seen at the Centre for Eye Health glaucoma service who had undergone frontloaded visual field tests (two tests per eye per visit). The frontloaded approach was originally studied in the Frontloaded Fields Study [9], which was then extended longitudinally as it became part of the clinical testing protocols. In brief, this meant that almost all patients seen within the clinical service routinely underwent two visual field tests per eye per clinical visit using the 24-2 SITA-Faster test on the Humphrey Field Analyzer (Carl Zeiss Meditec, zeiss.com/meditec-ag/en/home.html).

Default 24-2 SITA-Faster testing protocols were used: false positive catch trials and the gaze tracker were active, but fixation loss and false negative catch trials were not used. A white Goldmann size III stimulus with a stimulus duration of 200 ms was presented on a white background under low photopic conditions. Corrective lenses were used for testing as per the manufacturer’s recommendations. Test order (combinations of right eye trial 1, right eye trial 2, left eye trial 1 and left eye trial 2) was determined at the discretion of the perimetrist. Rest breaks were provided between tests as required.

Consecutive patients were reviewed for eligibility as participants for the present work. Inclusion criteria included: age >18 years, able to undertake perimetric testing and provided consent for use of their clinical data for research purposes. Specific diagnostic criteria were not required, nor were specific reliability criteria used at the beginning. For the purpose of the study, it was assumed that retest variability is likely to be related to the underlying sensitivity result, with the underlying pathophysiology (i.e., aetiology of loss) providing a minimal contribution. Instead, the study aimed to have a diversity of heterogeneous diagnoses to capture a spectrum of sensitivity values. Participants with a primary diagnosis of a significant media opacity or pre-retinal visual pathway disturbances (such as a dense cataract) were not included. A false positive catch trial (the default setting on SITA-Faster) cut-off value was not part of the inclusion criteria, as this was analysed as part of the study.

### Visual Field Data

A custom-written script in Python (Python Software Foundation, python.org/) was used to extract quantitative visual field data for all subjects. The present study was primarily interested in pointwise sensitivity. Although it is recognised that false positive rate cut-offs remain a topic of debate in the literature [10–12], this study continued to use the manufacturer-recommended cut-off value of ≤15% for inclusion. The presence of ‘Abnormally High Sensitivity’ glaucoma hemifield test results was also used as a method to exclude false positive results. As further sub-analyses, results were examined when using different false positive rate cut-offs.

### Quantification of the Measurement Floor and Clinically Meaningful Steps

Data from tests 1 and 2 were used as intra-visit test–retest data for quantitative analysis. Three methods were used to examine the steps.

### Method 1: Loess Functions—A Distribution-Based Approach

First, a method similar to that of Wall et al.’s approach was used [4]. All data (test 1 on the *x*-axis and test 2 on the *y*-axis) were plotted, and Loess functions were fitted to the 5th (lower) and 95th (upper) distributions at each test 1 sensitivity value. The Loess span (smoothing parameter) was set at 0.5, as a lower value may lead to overfitting. A line of unity (i.e., *x* = *y*) was added to the plot. Then, inspecting the figure from the right-hand side, the first instance of an intersection between the 95th percentile Loess function (the ‘upper Loess’) and the line of unity was marked. From this point, a vertical line was drawn downwards to intersect the 5th percentile Loess function (the ‘lower Loess’). The next step was defined by the horizontal line drawn from this intersection to the line of unity. This process was repeated until 0 dB was reached. Therefore, each horizontal span represented a range of test 1 ‘steps’, with the last step before 0 dB indicating an estimate of the measurement floor. Note that although the 5th percentile was taken, this was not a hypothesis test. Since tests 1 and 2 are inherently correlated repeated-measures, the use of the 5th percentile did not correspond with a compounded false positive probability of 0.25%.

The Loess approach is a distribution-based method for assessing variability and the dynamic range. The key advantage of using the 5th to 95th percentile range to represent the distribution of expected retest values is that it is relatively robust to outliers. In visualising data distributions, there are relatively fewer assumptions made about its behaviour. Conversely, this approach relies on the geometry of the Loess envelopes to infer the transition points.

### Method 2: Bland–Altman Analysis and Smoothed Loess Functions

One of the assumptions of the Loess function approach described by Wall et al. [4] is that the test–retest distribution is symmetric about the line of unity. However, since there are known differences between test 1 and test 2 when using intra-visit SITA-Faster visual fields [11, 13], this assumption may not hold with the present data. To examine this, the data were also plotted using the Bland–Altman approach. The same Loess smoothing function was used, and the 5th and 95th percentile range was determined. The same geometric step approach as per Method 1 was used to identify the steps on the Bland–Altman analysis. Note that a key difference was that the *x* values of a Bland–Altman plot represent the average of the input variable, and hence may not represent integer sensitivities. For clinical interpretability, the breakpoints (but not the raw data) were rounded to the nearest integer decibel value.

### Method 3: Gaussian Smooth Function and Multisegmental Linear Regression—A Parametric Approach

The third approach was a Gaussian smooth function and multisegmental linear regression. Here, the mean sensitivity was calculated for each test location for each subject, which served as the *x*-axis value (grouped into the nearest 1 dB bins for clinical interpretability). For the same test location, the standard deviation of test–retest pairs was also calculated, which served as the *y*-axis value. This resulted in a distribution of standard deviation values for each mean sensitivity bin. A 1D Gaussian filter (sigma = 1) was used to fit a smoothed curve to the test–retest standard deviation-mean sensitivity data. This was done to reduce potential noise that might confound the next step. Following this, a multisegmental linear regression function was fitted to the data to identify the breakpoints (*x*0) at which variance increases significantly.

The steps for multisegmental linear regression were as follows. A number between three and six segments was selected, each representing a combination of candidate breakpoints. The constraints were a minimum of 10 dB and a maximum of 35 dB. Similar to the Loess conditions, a minimum difference of 2 dB was required. Linear regression models were fitted to each segment, bounded by each breakpoint and assessed using least squares. The model was evaluated using the *R*^2^ value. A total of 10,000 models were assessed, and the model with the best fit quality was retained. Mathematically, the breakpoints represented a significant change in the rate of change in variance. This was chosen because variance is known to increase as visual field sensitivity decreases up to a critical point, before decreasing again. Therefore, changes in the variance trajectory may signify a transition towards another clinical step, and the last (smallest magnitude) breakpoint represents the measurement floor. This approach represents a different interpretation of the clinical step and was not considered equivalent to Methods 1 and 2. Given the expected inverted U-shaped function of variance as a function of sensitivity, the measurement floor was defined as the last breakpoint at which the rate of variance change continued to increase. In other words, the zenith of variance was not regarded as a breakpoint, as variance would have already been on a significant upward trajectory.

Unlike the geometric approach of Loess functions, the use of the multisegmental linear regression analysis provides a parametric method for quantifying potential breakpoints. Advantageously, this approach provides clearer clinical interpretability of the transition between sensitivity values with significantly different variances, whereby changes in values exceeding test–retest variability are often used as an indicator of clinical significance.

However, this method makes several assumptions. It presumes that standard deviation is a valid measure of test–retest variability and thus assumes symmetric scatter. This is a common assumption in perimetric research [14–16]. However, perimetric data is more likely asymmetric, heteroskedastic and have a directional regression to the mean, especially at higher values closer to normality or lower values close to the floor. This is due to the natural floor and ceiling values of perimetry. Applying a Gaussian kernel leads to smoothing of the standard deviation-mean sensitivity relationship, but this method assumes that it reduces noise without obscuring structural transitions. However, the purpose of the multisegmental analysis was to look for departures from homoscedasticity, i.e., the changes in variance trajectory. Therefore, this was considered to be a supplement to the preferred Loess approach described in Methods 1 and 2.

### Effect of Reliability on Perimetric Steps and the Measurement Floor

A false positive rate of 15% or less is one of the quantitative cut-offs for a reliable visual field test recommended by the manufacturer of the device. As mentioned above, this was used as a definition for a ‘reliable’ result. It was supplemented further with the exclusion of results with a Glaucoma Hemifield Test that reported ‘Abnormally High Sensitivity’. However, in addition to this criterion, dynamic range and the measurement floor outputs were also assessed when using no cut-off for false positive rate, i.e., inclusive of all data.

### Statistical Analysis

Detailed statistical analyses are shown in each methodological approach for determining the measurement floor and clinical steps. Descriptive statistics were used for the baseline characteristics of the subject cohort. Since the goal of the study was to characterise population-level sensitivity steps, no mixed models were applied to the data to account for potentially multiple data points contributed by each subject.

## Results

The full cohort comprised 9672 pairs of visual field tests from 1468 eyes of 748 subjects (368 males, 380 females; mean age 60.6 years with a standard deviation of 13.6 years). Using a false positive rate cut-off of 15% or lower resulted in 8460 pairs of visual field tests from 1333 eyes of 714 subjects (353 males and 361 females; mean age 60.8 years with a standard deviation of 13.6 years). Using a stricter criterion of 0% false positive rate resulted in 3923 test pairs from 944 eyes of 558 subjects (283 males and 275 females; mean age 61.8 years with a standard deviation of 13.1 years). Although not analysed as subgroups, the distribution of diagnostic groups were: 39.6% normal, 18.1% glaucoma suspect, 30.3% glaucoma, 10.5% non-glaucomatous optic nerve pathology (inclusive of retrograde degeneration) and 1.5% with non-optic nerve pathology.

**Fig. 1:**
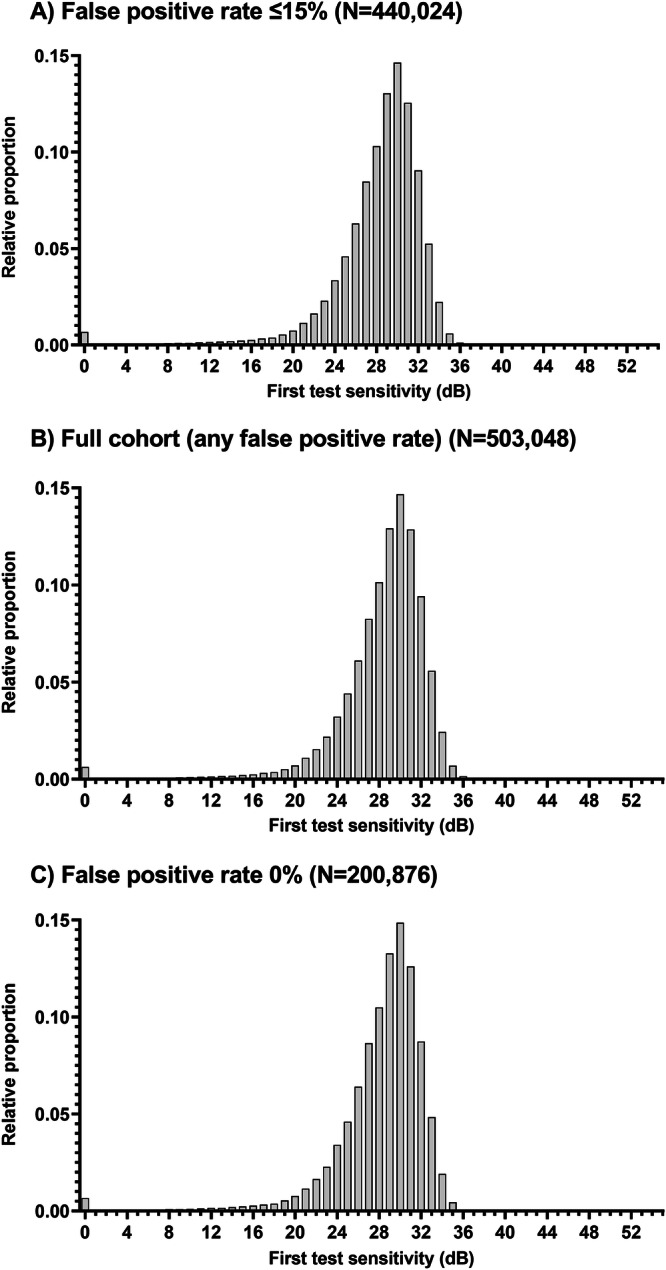
Distributions of first test sensitivity values (decibel, dB) for each false positive rate condition. **A** The subset of the cohort with false positive rate less than or equal to 15%; **B** The full cohort with any false positive rate; **C** The subset with a false positive rate of 0%. Relative proportions are used to demonstrate the shape of the distributions, due to unequal sample sizes, as described in the text. *N* shows the total number of individual test locations assessed.

The distributions of first test sensitivity values for each reliability criterion are shown in Fig. 1. In brief, the data demonstrated a right skew, with a peak sensitivity at 30 dB. The shapes of the distributions were similar across conditions, but the 0% false positive rate condition showed a slightly slimmer shape due to lower proportions of high sensitivity measurements. However, despite the right-sided skew, there were still 19,532, 21,247 and 9079 test pairs where the first sensitivity result was ≤20 dB for a false positive rate cut-off of ≤15%, total cohort and false positive rate 0% conditions, respectively.

### Quantification of the Number of Clinical Steps and Measurement Floor

**Fig. 2:**
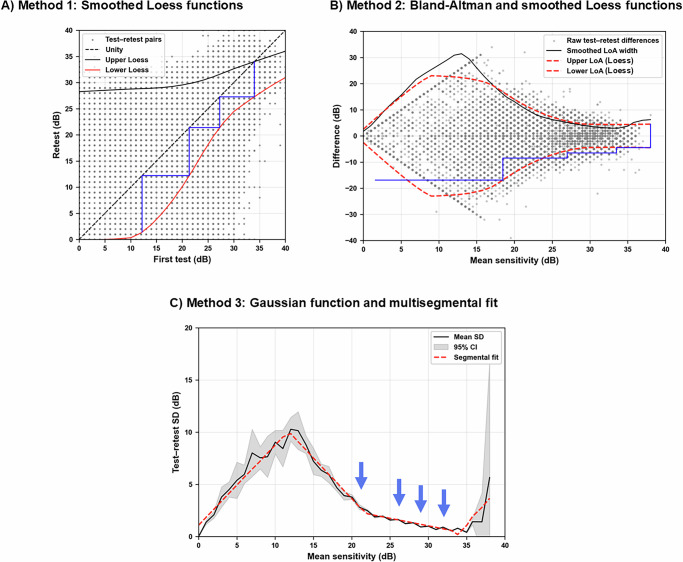
Each of the three methods used to examine the measurement steps (breakpoints and intervals) and floor for the reliability condition of false positive rate ≤15%. **A** The smoothed Loess functions (95th upper Loess, solid black; 5th lower Loess, solid red) are fitted to the data points. The black dashed line indicates the line of unity. The blue lines indicate each step and interval, as described in the ‘Methods’. **B** The same data in (**A**) but plotted on a Bland–Altman plot. Smoothed Loess functions (95th upper Loess, solid black; 5th lower Loess, solid red) are fitted to the data points, and the blue lines indicate each step and interval, as described in the ‘Methods’. The data points were reported as calculated, whilst the breakpoints were rounded to the nearest integer. **C** The standard deviation (SD) of test–retest values at each mean sensitivity bin (dB). The mean of the SD value at each bin is shown in the black line, and the 95% confidence interval is shown in grey. The red dashed line indicates the multisegmental fit to highlight a breakpoint, with the blue downward arrows indicating the breakpoints in the multisegment regression where the trajectory of variance changes significantly, as described by the ‘Methods’. As noted in the ‘Methods’, changes beyond the uptick at 35 dB and below the zenith of variance were not regarded as breakpoints. dB decibel.

**Fig. 3:**
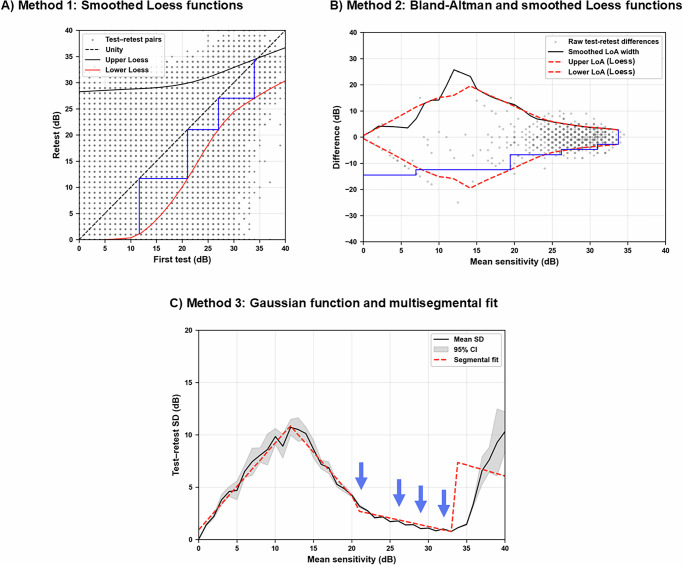
Each of the three methods used to examine the measurement steps (breakpoints and intervals) and floor for the reliability condition of any false positive rate. The plots are as described in the Fig. 2 caption. As mentioned in the text, **B** contains the data for test locations at eccentricity >16° (see text and Supplementary Material) to illustrate the Loess functions. dB decibel.

**Fig. 4:**
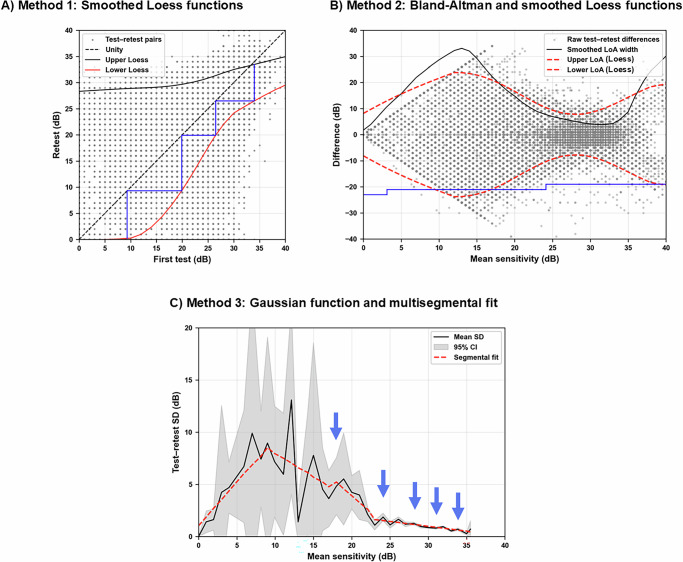
Each of the three methods used to examine the measurement steps (breakpoints and intervals) and floor for the reliability condition of false positive rate equals 0%. The plots are as described in the Fig. 2 caption. dB decibel.

The results for Methods 1, 2 and 3 applied to the cohort with a ≤15% false positive rate, the complete cohort and the subgroup with a false positive rate of 0% are shown in Figs. 2, 3 and 4, respectively. The quantitative number of clinical steps, intervals and measurement floor values are summarised in Table 1.

**Table 1:** Number of clinical steps, breakpoints and measurement floor estimations for each method in the present study.

	Number of clinical steps	Breakpoints between steps	Measurement floor
Method 1: Smoothed Loess functions			
False positive rate ≤15%	4	34, 27, 21	21
Total cohort	4	34, 27, 21	21
False positive rate 0%	4	34, 27, 20	20
Method 2: Bland–Altman with Loess functions			
False positive rate ≤15%	4	33, 27, 18	18
Total cohort^a^	n/a	n/a	n/a
False positive rate 0%^b^	4	31, 26, 19	19
Method 2: Gaussian function and multisegmental fit			
False positive rate ≤15%	5	32, 29, 26, 21	21
Total cohort	5	32, 29, 26, 21	21
False positive rate 0%	6	34, 31, 28, 24, 18	18

^a^The steps were not valid for this condition.

^b^These results are for the eccentricity >16° condition, due to the flattened Loess function when using the full data set.

Figures 2–4 show similar patterns in the change in magnitude of test–retest variability. Several findings were evident when comparing the false positive rate conditions. First, the Loess method (Method 1; Figs. 2–4, panel A) returned similar results across the three false positive rate conditions. There were consistently four sensitivity intervals, with similar measurement floor values (21, 21 and 20 dB) for false positive rate ≤15%, all results and false positive rate 0%, respectively. Method 1 also returned a similar total range across the reliability conditions (13–14 dB).

The second approach, using Bland–Altman plots and the same Loess functions (Figs. 2–4, panel B), resulted in the same number of steps for the false positive rate ≤15% and 0% conditions. The breakpoints were also similar for the false positive rate ≤15% condition, but the upper bracket was notably different for the false positive rate 0% condition. Notably, for the false positive rate 0% condition, the sampling on the left side of the Bland–Altman plot was relatively sparse, with a tendency towards a test 1-test 2 difference of zero. This flattened the Loess functions, resulting in no geometric steps. Therefore, for representative purposes, the >16° eccentricity data were used for this condition to illustrate the fitted Loess functions. The total cohort condition did not return valid steps due to the profound spread of data.

The third approach used Gaussian functions that were fitted with multisegmental regression (Figs. 2–4, panel C). The breakpoints represent a difference in the change in variance, indicated by the downward blue arrows. The increased granularity of segmental line fits resulted in one additional breakpoint in comparison with the Loess envelopes for the false positive rate ≤15% and total cohort conditions. Although the increased granularity led to more breakpoints for the false positive rate ≤15% and total cohort conditions, the total decibel range between the highest and lowest breakpoints (11 dB for both) was lower compared to Method 1. The false positive rate 0% condition had a further additional breakpoint (five in total), with a lower floor of 18 dB and a wider decibel range of 16 dB. As mentioned, the floor was the last breakpoint at which variance continued to accelerate and was not the zenith of variance magnitude. Although the variance below 18 dB was expectedly high and aligned with expectations, there were also wide distributions of variance between the floor and the penultimate step (i.e., 18 dB and 24 dB). This was likely a product of lower sampling due to the stringent false positive rate requirement.

### Effect of Eccentricity on Discernible Steps and the Measurement Floor

The 24-2 test locations were separated into three approximate eccentricity ranges: within 10° of fixation, 10–16° from fixation and >16° from fixation (with focus on the cohort with a false positive rate ≤15%). Although there were subtle differences in the breakpoints, intervals and measurement floor, the issue of using these eccentricity ranges was that the distribution of initial sensitivity value became skewed. For example, there was relatively less representation of lower sensitivity within the central 10° from fixation subgroup, potentially explaining the increased sensitivity level for its brackets and floor. Conversely, by definition, there were fewer high sensitivity values and more lower sensitivity values in the >16° subgroup, leading to slightly lower intervals and floors. Since the eccentricity-based differences are likely due to surrogate effects of expected sensitivity ranges, these are shown in Supplementary Material.

## Discussion

Using a conventional reliability criterion of false positive rate ≤15%, small differences in results were found across methods, with four to five calculable intervals and a measurement floor of 19–21 dB. Using a more stringent reliability criterion of a false positive rate 0% resulted in slightly more intervals and a lower measurement floor of 18–20 dB. Analysis of the full cohort was less reliable, with a skew towards higher sensitivity values for the intervals.

### Effective Dynamic Range in Perimetric Testing

Wall et al. [4] first defined the effective dynamic range of visual field testing and compared the number of clinically-relevant steps and measurement floor using four different perimetry approaches. They found that visual field testing using the SITA-Standard algorithm and a Goldmann size III stimulus returned four intervals and a measurement floor of either 15 dB or 19 dB when using criteria of a most frequent retest value of 0 dB or the threshold at which retest estimates were greater than 5% of the floor effect.

Using a similar approach of fitted Loess functions, the present study also found four discernible intervals when using SITA-Faster visual field tests. However, the current ‘floor’ criterion differs slightly, whereby very small non-zero thresholds were accepted to be effectively the same as 0 dB. For example, the ‘step’ ended at 2 dB for the false positive rate ≤15% cohort, as it was deemed unlikely that an interval of 0-2 dB is clinically meaningful.

However, an issue with the Loess approach on a conventional *x*–*y* plot is that it assumes a symmetric distribution in test–retest variability, and therefore, the valid use of the line of unity as a reference point. As previously noted, SITA-Faster, especially when performed on the same day, has a systematic difference between test 1 and test 2, with test 2 more likely to return more reliable results [13]. As such, although the *x*–*y* plot potentially shows a more pragmatic result, the Bland–Altman approach can provide a more balanced and symmetric view of test–retest differences.

Despite its assumptions, the multisegmental function applied to the Gaussian smoothed standard deviation-mean sensitivity relationship allowed the detection of subtle changes in variance across sensitivity bins. This was aimed at addressing the clinical question of the limits at which changes in sensitivity coincide with significant differences in variance, and thus potentially clinically significant differences. The changes occurred mainly in the mid-to-upper sensitivity range. The uptick of variance in sensitivity values >35 dB has been shown previously with SITA-Faster [17]. Due to the nature of the multisegmental regression analysis, it was not treated as a separate interval; instead, the interval started from 32 dB onwards. From a clinical perspective, a 35 dB result is likely present within the central 5° of the visual field and in the 24-2 test grid, represented by only four test locations. Thus, the number of discernible intervals between a mid-30s sensitivity and the measurement floor is likely more important than quantifying different levels of relatively higher sensitivity.

Later, Gardiner [18] described upper and lower bounds of perimetry to be 17–31 dB. Whilst the lower and upper bounds in the current results were slightly higher, the overall range of values (i.e., the full range in the dynamic range) was similar at 13–14 dB when using Method 1, with more variable results for Method 2 (11–16 dB). In agreement with Gardiner [18], the present results show a very narrow range of clinically useful sensitivity outputs in perimetry.

### The Measurement Floor in Perimetry

Gardiner et al. [5] correlated the frequency of seeing curves and perimetric sensitivity results, and defined the lower limit of reliable testing as the point at which further contrast changes did not change the response rate. This was approximately 15–19 dB, consistent with the work of Wall et al. [4]. This value was based on the relationship between the Method of Constant Stimuli and perimetric sensitivity measurements deteriorating significantly at 15–19 dB. Another criterion is whether there is a statistically significant (despite a low coefficient of determination) relationship between the Method of Constant Stimuli and perimetric sensitivity (18 dB). Alternatively, defining the floor as the highest perimetric sensitivity value at which the probability of detection fell just below 50% led to a result of 20.5 dB.

In the present study, different methodologies and cohorts led to slight differences in the measurement floor, consistent with the work of Gardiner et al. [5]. However, for a commonly used reliability criterion of false positive rate ≤15%, the measurement floor values were consistently 21 dB for both methods. Therefore, the present results returned a measurement floor that was slightly higher than that of both Wall et al. [4] and Gardiner et al. [5]. Whilst a difference in cohort or methodological approach may account for the difference, it is also possible that the use of SITA-Faster may have led to a slightly higher floor in the present study. As per our hypothesis, the higher variability in SITA-Faster measurements, in comparison to full threshold and SITA-Standard (which were used in previous work), may lead to a higher floor. Another reason is the tendency for SITA-Faster to return slightly higher sensitivity values, on average, compared with previous generations of SITA [8, 17]. This is an especially important consideration if switching between algorithms [7, 19, 20].

Practically, the measurement floor represents the approximate threshold at which progression no longer changes meaningfully. Previous studies [21, 22] on censorship of test location reaching the measurement floor propose methods for avoiding spurious changes and improving test efficiencies by regarding those test locations as defective with no further requirement for repeated testing. Another practical consideration is the transition to other test grids, such as using the 10-2 test grid [23–25] and/or the use of a Goldmann size V stimulus [26, 27], which could extend the dynamic range [28]. The rationale behind a 10-2 or equivalent test grid is not necessarily related to the difference in eccentricity, but instead the density of testing, which allows for more test locations to be examined and potentially return more clinical information. Larger stimuli, such as a Goldmann size V, have been shown to return similar benefits due to decreased variance and greater effective energy delivery.

### Effect of Measurement Reliability on the Effective Dynamic Range

Previous studies have reported higher false positive rates when using SITA-Faster in comparison with previous generations of SITA, which have led to concerns of greater rates of low test reliability [8, 12, 17]. However, the elevations in false positive rate have later been shown to have poor associations with other metrics of test reliability and with key resultant perimetric indices [10, 11]. Accordingly, this has led to suggestions that the 15% false positive rate cut-off may be unnecessarily stringent for clinical purposes.

Although issues were anticipated with using a more inclusive inclusion criterion from a false positive rate perspective due to the expansion of the range of possible test–retest values, all methods returned results that were similar when comparing the full cohort and the subset with false positive rate less than or equal to 15%. This suggests that, at the group level, false positive rates minimally affect the measurement step intervals of the measurement floor. However, in the context of previous work on false positive rates, clinicians are urged to interpret retest sensitivity with caution, especially where there are different false positive rates between tests. Thus, interpretation of pointwise sensitivity change requires the combination of the intervals of discernible change alongside differences in false positive rates (or, in general, reliability) between tests. 

In comparison, the stricter reliability criterion of a false positive rate of 0% resulted in a lower measurement floor (20 dB for Method 1, 19 dB for Method 2 and 18 dB for Method 3). This also generally resulted in a greater number of discernible measurement steps than the other criteria, likely attributable to the overall lower variability. However, the concern with the stringency of this criterion is the much lower sampling, especially at lower sensitivities, which confounds analysis.

### Limitations

The current large sample size of visual field findings only included results obtained using SITA-Faster, and thus, different SITA test algorithms could not be compared directly. Rather, previous studies were relied upon, which have documented different variability characteristics comprehensively for the SITA family. Also, multiple results were used from the same subject. Since correlations were not examined directly, this was not corrected for. Further, it was assumed that the main contributor to the test–retest analysis was the underlying sensitivity. The primary diagnosis was used for separating diagnostic groups, and sub-analyses of specific causes of sensitivity loss (such as outer versus inner retinal pathology) were not performed. In addition, patients with significant pre-retinal loss were not included. This could be explored in future studies.

Paired test results were obtained at the same clinical visit. This has the advantage of reducing the contribution of potential progression or inter-test learning. Although performed during the same visit, SITA-Faster has been shown to have minimal issues related to fatigue due to its short test time. A caveat to the current findings is that intra-test variability may differ from inter-test variability. Our recent results have suggested that SITA-Faster, at least for follow-up visits within a year, tend to have similar test–retest characteristics both within and across visits [12]. Furthermore, tests that are separated by longer time intervals tend to have more volatility with respect to variability estimation, due to the need to incorporate progression information [16]. Short-term testing tends to return more stable estimates of variability [16]. Therefore, these results reflect a situation in which test–retest variability does not account for the contributions from progression and temporal separation.

Due to the nature of the cohort, there was a proportionally lower sampling of low sensitivity values, especially when the analysis was further divided by eccentricity, as flagged by Gardiner [5, 29]. Subtle differences were found (but with differences of 1 dB, unlikely to be clinically significant), which are likely to be accounted for by the imbalance in sensitivity sampling at different eccentricities. Nonetheless, across the whole cohort, over 20,000 data points were analysed near the floor, which should still provide a robust estimation of nearby breakpoints. Future studies could explore this further.

## Conclusions

SITA-Faster returned a similar number of discernible sensitivity intervals compared to full threshold and SITA-Standard, but with a slightly higher measurement floor. This reflects the slightly higher returned sensitivity values of SITA-Faster, its higher variability and its algorithmic treatment of low sensitivity thresholds. Clinicians should adjust their expectations for meaningful perimetric change when interpreting SITA-Faster visual field results. Practically, extensive loss could prompt the transition to alternative testing methods, such as the 10-2 test grid and/or larger stimuli such as a Goldmann size V.

## Supplementary Information

Supplementary Material

## Data Availability

The data that support the findings of this study are not openly available due to reasons of sensitivity and are available from the corresponding author upon reasonable request.
