# Supplementary material for: Clinical Effective Dynamic Range and the Measurement Floor of SITA-Faster Visual Field Tests
Source: Ophthalmic Physiol Opt. 2026 May 11;46(4):991–9. doi: 10.1007/s44402-026-00103-6 (PMC13395987; doi:10.1007/s44402-026-00103-6)
Supplement: Supplementary file 1 — Supplementary Material [file 44402_2026_103_MOESM1_ESM.docx]

**Supplementary Material: Clinical effective dynamic range and the measurement floor of SITA-Faster visual field tests**

Jack Phu^1,2^, Henrietta Wang^1^, Michael Kalloniatis^1,2,3^

1. School of Optometry and Vision Science, University of New South Wales, Kensington, New South Wales, Australia
2. University of Houston College of Optometry, Houston, TX
3. School of Medicine (Optometry), Deakin University, Waurn Ponds, Victoria, Australia

Number of Figures: 4 Number of Tables: 1

Corresponding Author: Jack Phu

Address for reprints: School of Optometry and Vision Science, Gate 14 Barker Street Rupert Myers Building South Wing, University of New South Wales Sydney 2052, New South Wales, Australia

Email: [jack.phu@unsw.edu.au](mailto:jack.phu@unsw.edu.au)

*Keywords*: visual fields; perimetry; standard automated perimetry; 24-2; frontloaded

Financial support: The work was supported, in part, by an NHMRC Ideas Grant to MK and JP (1186915). The funding organisation had no role in the design or conduct of this research.

Conflict of interest: No conflicting relationship exists for any author.

Data availability statement: The data that support the findings of this study are not openly available due to reasons of sensitivity and are available from the corresponding author upon reasonable request.

Author contributions: All authors contributed to the study conception and design. Material preparation and data collection were performed by JP and HW, and analysis was performed by JP, HW and MK. The first draft of the manuscript was written by JP and all authors commented on previous versions of the manuscript. All authors read and approved the final manuscript.

Running head: Intrinsic variability in perimetry

| Supplementary Table 1: Number of clinical steps, breakpoints and measurement floor estimations for three eccentricity rings under the condition of false positive rate <=15% in the present study. | | | |
| --- | --- | --- | --- |
|  | Number of clinical steps | Breakpoints between steps | Measurement floor |
| Method 1: Smoothed Loess functions | | | |
| Inner 10 degrees | 4 | 34, 27, 22 | 22 |
| Middle 10-16 degrees | 4 | 34, 27, 20 | 20 |
| Outer >16 degrees | 4 | 32, 25, 19 | 19 |
| Method 2: Gaussian function and multisegmental fit | | | |
| Inner 10 degrees | 6 | 33, 31, 29, 26, 22 | 22 |
| Middle 10-16 degrees | 5 | 32, 29, 26, 21 | 21 |
| Outer >16 degrees | 6 | 34, 31, 28, 24, 19 | 19 |


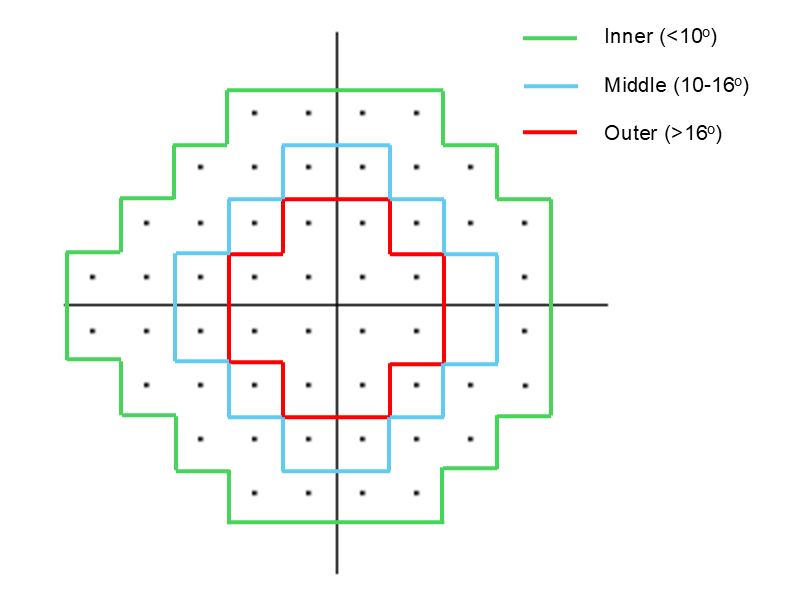


Supplementary Figure 1: The three nominal eccentricity rings used for eccentricity-based analysis. The inner ring is defined as within 10 degrees of fixation (red outline). The next ring, the middle, is defined as 10-16 degrees from fixation (blue outline). The outer ring is defined as >16 degrees from fixation (green outline).


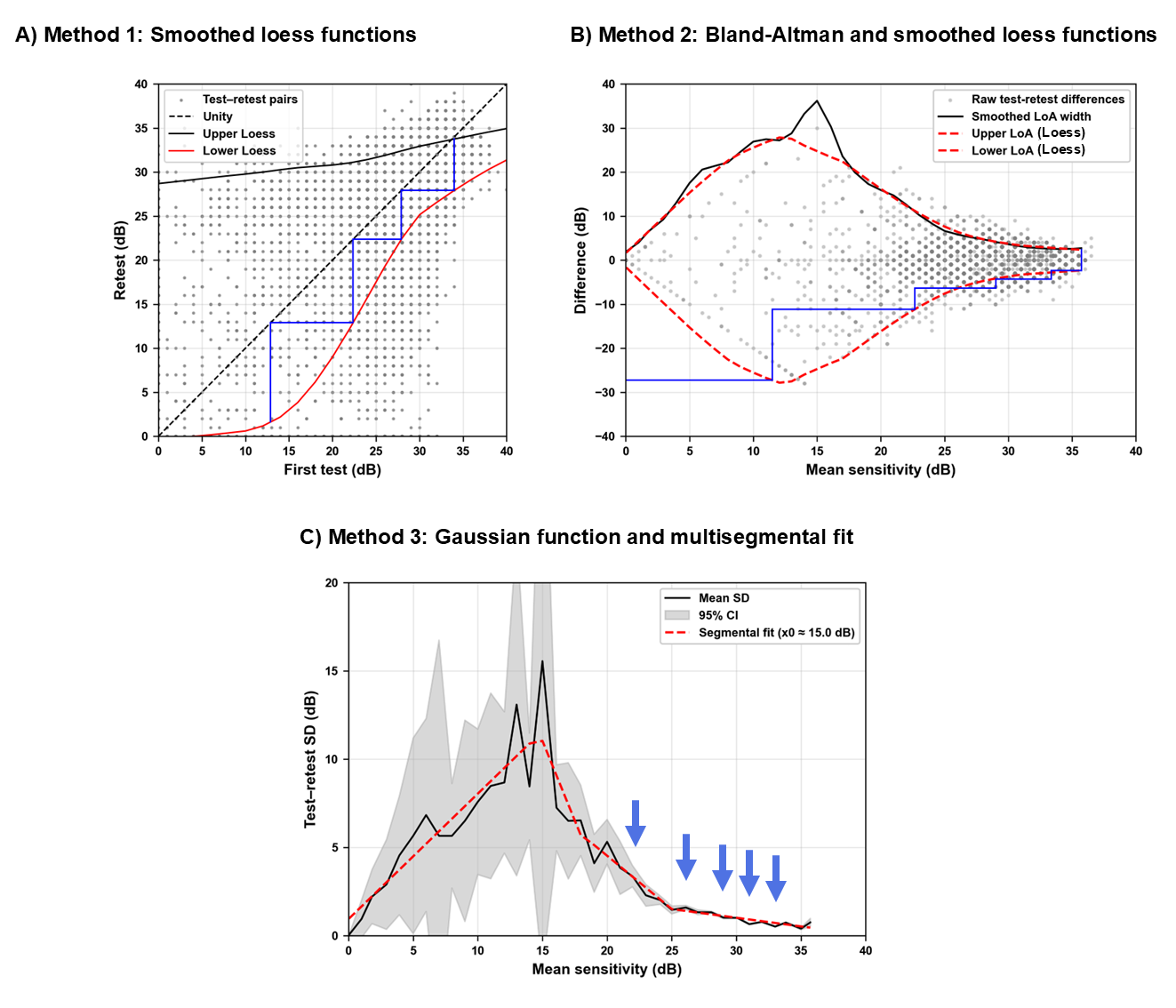


Supplementary Figure 2: Each of the two methods used to examine the measurement steps (breakpoints and intervals) and floor for the reliability condition of false positive rate ≤15% for the inner ring (as per Supplementary Figure 1). A) The smoothed Loess functions (95^th^ upper Loess, solid black; 5^th^ lower Loess, solid red) are fitted to the data points. The black dashed line indicates the line of unity. The blue lines indicate each step and interval, as described in the Methods. B) The same data in A) but plotted on a Bland-Altman plot. Smoothed Loess functions (95^th^ upper Loess, solid black; 5^th^ lower Loess, solid red) are fitted to the data points, and the blue lines indicate each step and interval, as described in the Methods. C) The standard deviation (SD) of test-retest values at each mean sensitivity bin (dB). The mean of the SD value at each bin is shown in the black line and the 95% confidence interval is shown in grey. The red dashed line indicates the multisegmental fit to highlight a breakpoint, with the blue downward arrows indicating the breakpoints as described by the Methods.


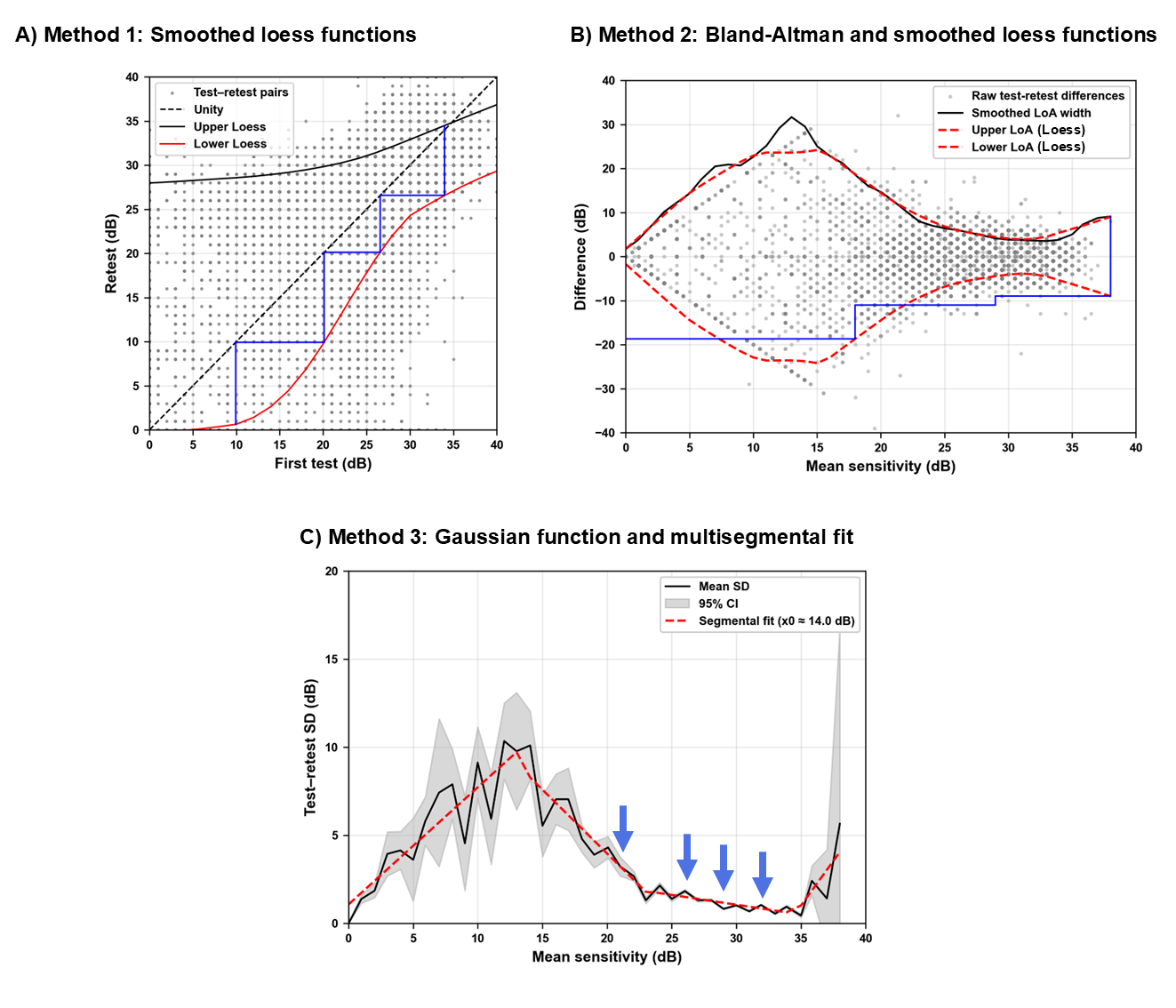


Supplementary Figure 3: Each of the two methods used to examine the measurement steps (breakpoints and intervals) and floor for the reliability condition of false positive rate ≤15% for the middle ring (as per Supplementary Figure 1). The plots are as described in the Supplementary Figure 2 caption.


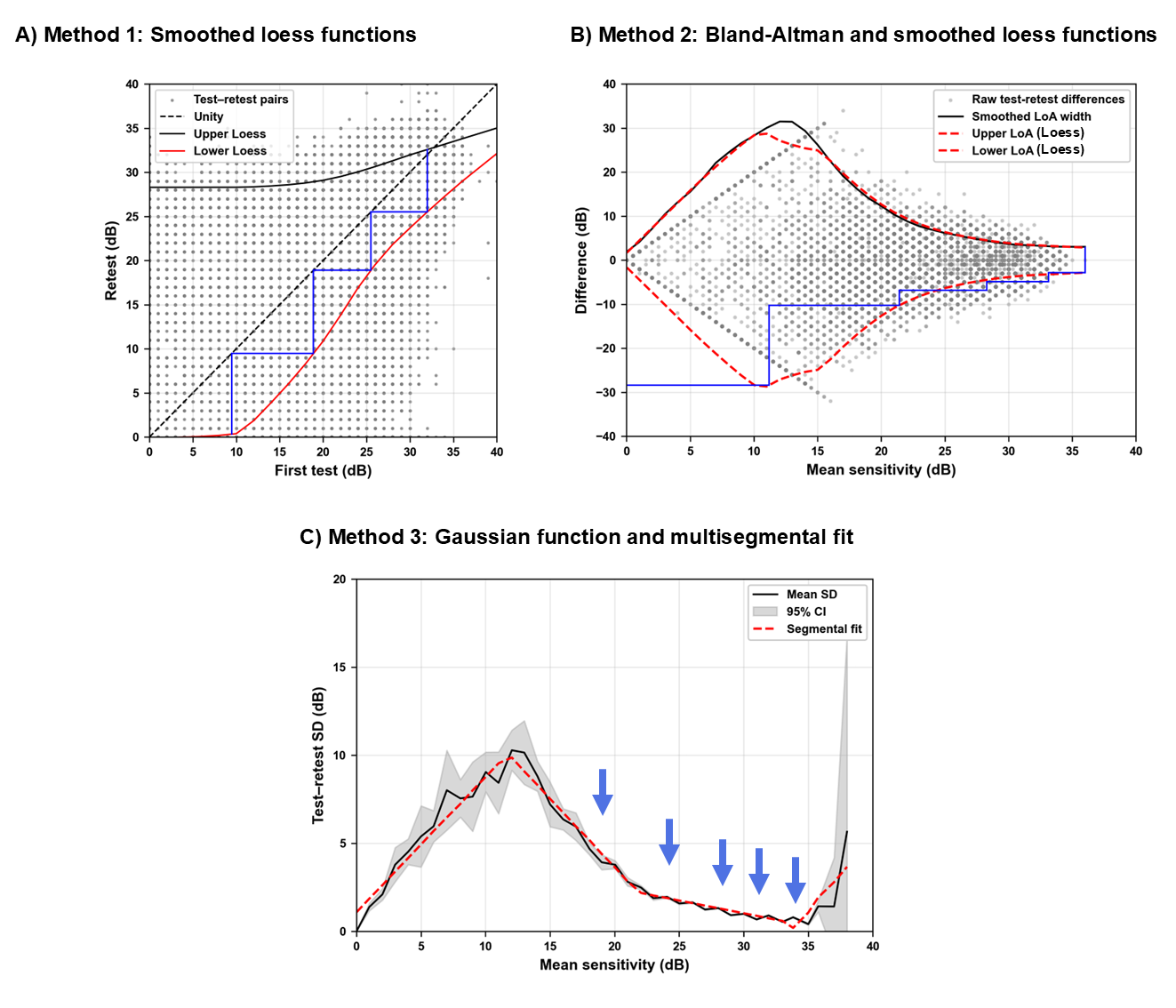


Supplementary Figure 4: Each of the two methods used to examine the measurement steps (breakpoints and intervals) and floor for the reliability condition of false positive rate ≤15% for the outer ring (as per Supplementary Figure 1). The plots are as described in the Supplementary Figure 2 caption.
